# Regional Odontodysplasia in Primary Teeth: A Case Report

**DOI:** 10.7759/cureus.85149

**Published:** 2025-05-31

**Authors:** Thaís Kaline O Souza, Annelyze P Kloster, Luiz E Volpato, Lorraynne S Lara

**Affiliations:** 1 Pediatric Dentistry, Private Practice, Peixoto de Azevedo, BRA; 2 Dentistry, Centro Universitário de Várzea Grande, Várzea Grande, BRA; 3 Dentistry, Hospital de Câncer de Mato Grosso, Cuiabá, BRA; 4 Graduate Program in Integrated Dental Sciences, Universidade de Cuiabá, Cuiabá, BRA

**Keywords:** child oral health, conservative treatment, deciduous dentition, developmental disorders, odontodysplasia, pediatric dentistry, tooth abnormalities

## Abstract

Regional odontodysplasia (RO) is a rare dental anomaly that affects the development of dental tissues, resulting in amorphous, hypocalcified, hypoplastic teeth with the characteristic radiographic appearance of “ghost teeth.” This study aims to report the clinical case of a patient diagnosed with RO in primary teeth. A two-year-old boy was brought to a private clinic with complaints of delayed eruption and anomalies in the upper right primary teeth. Intraoral examination revealed significant changes in the shape, color, and structure of all upper right primary teeth, including enamel hypoplasia, conoid and flattened teeth, yellowish/brownish coloration, and associated gingival inflammation. Radiographic examination revealed a thin layer of enamel and dentin, with no contrast between them, a pattern characteristic of “ghost teeth.” A conservative approach was adopted, including prophylaxis, application of fluoride varnish, guidance, and regular clinical and radiographic follow-up, aiming to maintain the health of the affected tissues and monitor the development of the permanent dentition. This case highlights the importance of early recognition of RO, which enabled an individualized treatment plan focused on preserving the affected teeth and promoting oral health.

## Introduction

Regional odontodysplasia (RO) is a rare, nonhereditary, localized, and complex condition that affects dental tissues derived from both ectodermal and mesodermal origins [1,2]. Its etiology remains unclear, although factors such as viral infections, local trauma, hyperpyrexia, teratogenic drug use during pregnancy, radiation exposure, nutritional and metabolic disorders, vitamin deficiencies, Rh incompatibility, and genetic syndromes have all been associated with its development [1-6].

There is no evidence of ethnic predisposition to RO [2,3]. Some studies suggest a higher prevalence in female patients [2,4,5], although this remains controversial [1]. The condition can affect both the primary and permanent dentitions [1,5,6], with the maxilla being affected approximately twice as often as the mandible [1,2,7]. Although it is usually confined to a single quadrant [1,4,6], RO may affect just one tooth or, in rare cases, present in a generalized form [1,8].

Clinically, teeth affected by RO are amorphous, undersized, grooved, hypocalcified, and hypoplastic. Crowns are typically short, with an irregular shape and a yellow to brown coloration [1,2,4]. The enamel is softened, and the affected teeth are highly susceptible to caries [9]. Recurrent infections and tooth mobility are also common [1,10]. Radiographically, RO presents as a thin, poorly defined layer of enamel and dentin, lacking contrast, which gives the affected teeth their characteristic “ghost-like” appearance [3,4]. The pulp chambers are often enlarged, with short roots and open apices [1,4,6].

Diagnosis is primarily based on clinical and radiographic findings, and early identification is crucial for treatment planning and adequate follow-up [2,3,10]. Given the rarity of this anomaly, the limited number of studies, and the importance of an early approach, this report presents a clinical case of a patient diagnosed with RO in the primary dentition.

## Case presentation

A two-year-old boy presented to a private dental clinic accompanied by his mother, who reported delayed eruption of the upper right primary teeth. According to her, when the teeth began to erupt, they appeared abnormal in shape and color. The mother reported regular prenatal care with no complications and normal examination results. The birth was full-term and uneventful, and the neonatal period progressed without issues. The mother has no history of diseases or significant medical events during pregnancy. There is no family history of similar conditions or hereditary disorders.

**Figure 1:**
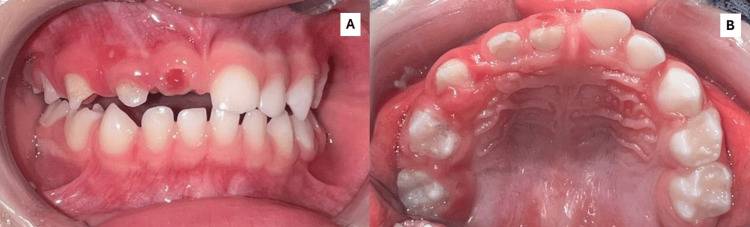
Intraoral view showing the dental arches. (A) Frontal view highlighting tooth 51 with a reduced crown, yellow-brown enamel, irregular surface, and associated gingival inflammation; tooth 52 with a short crown, yellow enamel with hypoplasia, and an ulcerated lesion on the adjacent gingiva; tooth 53 partially erupted with an irregular, shortened, flattened, and conoid crown, enamel hypoplasia, and yellow-brown discoloration; teeth 54 and 55 partially erupted, with undefined contours, amorphous appearance, opaque enamel, and enamel absence in some areas. (B) Occlusal view of the maxillary arch showing structural alterations in the right-sided teeth, with enamel hypoplasia, irregular crown contours, and yellow to brown discoloration. The left-sided teeth have preserved shape, contour, and color, with an eruption pattern consistent with the patient’s age. The clinical features are compatible with a diagnosis of regional odontodysplasia

Extraoral examination revealed no abnormalities. Intraoral examination showed that the patient was in the primary dentition stage, with all lower teeth and upper left teeth appearing normal. However, the upper right primary teeth exhibited multiple anomalies: the central incisor had a reduced crown size and yellow-brown enamel; the lateral incisor had a short crown, yellowish color, and signs of enamel hypoplasia; the canine, not fully erupted, showed a conoid-shaped crown with yellow-brown discoloration, hypoplastic enamel, and a shortened, flattened shape; the first molar was partially erupted, reduced in size, and amorphous in appearance, with an opaque and yellowish occlusal surface; the second molar was also partially erupted, with a flattened crown, enamel loss in some areas, intense yellow discoloration, and an undefined occlusal contour. The gingiva associated with these teeth was hyperplastic and reddish, suggestive of localized inflammatory gingival overgrowth (Figure 1).

**Figure 2:**
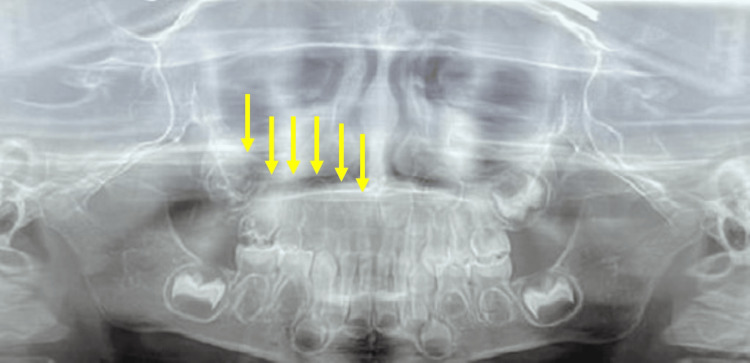
Panoramic radiograph showing the involvement of the upper right primary teeth, with reduced radiopacity, poorly defined crown contours, enlarged pulp chambers, and short or absent roots, consistent with the “ghost tooth” pattern. The germs of the upper right permanent teeth are either absent or show underdevelopment. In contrast, the upper left and lower primary teeth exhibit root and crown development appropriate for the patient’s age

Radiographic examination showed reduced thickness and radiodensity of the enamel and dentin in the upper right primary teeth, consistent with the “ghost tooth” pattern. These teeth displayed radiolucent crowns with irregular contours, enlarged pulp chambers, and short, poorly defined roots. The permanent tooth germs on the upper right side were either absent or underdeveloped. In contrast, the upper left and lower primary teeth showed root and crown development compatible with the patient's age, with no structural alterations, confirming the regional pattern of odontodysplasia (Figure 2).

Based on the clinical and radiographic findings, a diagnosis of RO was established. The mother was informed about the condition, the poor prognosis of the dysplastic teeth, and the importance of periodic dental monitoring. The initial treatment plan involved prophylaxis to control bacterial biofilm, which was causing gingival inflammation, and to minimize the risk of caries development. Topical fluoride varnish was applied to aid in the remineralization of the remaining enamel. Oral hygiene instructions were provided to the mother. The patient will remain under periodic clinical and radiographic follow-up to maintain oral health and monitor the development and eruption of the permanent teeth.

## Discussion

We present a case of RO in a two-year-old male child, emphasizing the importance of early diagnosis, which enabled a conservative management approach. RO is a rare developmental dental condition that can affect both the primary and permanent dentitions [2,4,9]. Among patients diagnosed with RO, exclusive involvement of primary teeth occurs in 5%-19.3% of cases, while only permanent teeth are affected in 28.9%-34.2%. Involvement of both dentitions has been observed in 46.6%-66.1% of cases [1,6]. Therefore, early diagnosis allows for an approach focused on preserving the primary teeth and monitoring the development of the permanent teeth [11].

Although some studies have reported a higher prevalence of RO in female patients, with ratios ranging from 1.37:1 to 1.7:1 [2,4,5,7,9], a recent systematic review of case reports found no significant sex difference. The authors suggest that this discrepancy may be due to a recent increase in case reporting, potentially reflecting a publication bias in earlier studies or improved diagnostic accuracy over time [1].

Most RO cases involve only one quadrant of the dental arch [4,6,9,10], with a preference for the maxilla [1,2,5,6,10], and the left side [1,2,6]. The right maxillary quadrant is the second most frequently affected [6]. In the present case, the restriction to the right maxillary quadrant reinforces the localized nature of the condition and aligns with previously documented presentation patterns. Although the exact reason for the preferential involvement of certain quadrants remains unclear, this asymmetric prevalence suggests a nonsystemic etiology and indicates that distinct etiological factors may be involved in each case [1,6]. In this context, early traumatic events, such as local trauma at two months of age [12] or maternal trauma during the third trimester of pregnancy, which could result in RO as the sole identified consequence [13], are notable considerations.

The diagnosis was based on the patient's medical history, including the nonhereditary nature of the condition, irregular tooth morphology, yellow-to-brown discoloration, enamel hypoplasia, and delayed eruption affecting a single quadrant, in association with inflammatory gingival hyperplasia [3]. Radiographic analysis revealed marked hypoplasia and decreased radiodensity of enamel and dentin, enlarged pulp chambers, and malformed, short roots [1,3,4,6], confirming the clinical suspicion of RO, along with the absence or underdevelopment of the permanent tooth germs.

Although the prognosis for RO is generally unfavorable, treatment depends on the teeth involved, the severity of the dysplasia, the presence of infection, and the patient’s age [13]. In this case, early diagnosis allowed for a conservative approach involving prophylaxis and fluoride varnish application, aiming to preserve dental structures, control mineral loss, and reduce sensitivity [14,15].

Management of RO should be individualized, as there is no standardized therapeutic protocol due to the rarity and clinical variability of the condition [2,6,13]. In cases identified early, as in the present report, conservative management is recommended to preserve the affected dentition and monitor dental and skeletal development [11]. On the other hand, in cases of late diagnosis or when recurrent infections are present, invasive interventions such as extractions or prosthetic rehabilitations may be necessary [10,11]. In the present case, considering the patient’s young age, the presence of primary dentition only, and the restriction of the condition to the right maxillary quadrant, the management strategy aimed to preserve the patient’s oral health until the eruption of the permanent teeth. Clinical and radiographic follow-up is crucial to promptly detect any manifestations in the permanent dentition.

This case highlights the relevance of early diagnosis and individualized treatment planning, with an emphasis on conservative management to preserve the dentition and maintain the patient’s quality of life.

## Conclusions

The presented case illustrates the classic clinical and radiographic features of RO in the primary dentition, emphasizing the importance of early diagnosis for a conservative management approach that aims to preserve the affected primary teeth and the patient’s quality of life, while also raising parental awareness regarding the need for follow-up of the development and eruption of the permanent dentition.
